# Transcriptome analysis of woodland strawberry (*Fragaria vesca*) response to the infection by *Strawberry vein banding virus* (SVBV)

**DOI:** 10.1186/s12985-016-0584-5

**Published:** 2016-07-13

**Authors:** Jing Chen, Hanping Zhang, Mingfeng Feng, Dengpan Zuo, Yahui Hu, Tong Jiang

**Affiliations:** School of Plant Protection, Anhui Agricultural University, Hefei, 230036 People’s Republic of China

**Keywords:** Woodland strawberry (*Fragaria vesca*), *Strawberry vein banding virus*, Transcriptome analysis, Pathogenic mechanism

## Abstract

**Background:**

Woodland strawberry (*Fragaria vesca*) infected with *Strawberry vein banding virus* (SVBV) exhibits chlorotic symptoms along the leaf veins. However, little is known about the molecular mechanism of strawberry disease caused by SVBV.

**Methods:**

We performed the next-generation sequencing (RNA-Seq) study to identify gene expression changes induced by SVBV in woodland strawberry using mock-inoculated plants as a control.

**Results:**

Using RNA-Seq, we have identified 36,850 unigenes, of which 517 were differentially expressed in the virus-infected plants (DEGs). The unigenes were annotated and classified with Gene Ontology (GO), Clusters of Orthologous Group (COG) and Kyoto Encyclopedia of Genes and Genomes (KEGG) analyses. The KEGG pathway analysis of these genes suggested that strawberry disease caused by SVBV may affect multiple processes including pigment metabolism, photosynthesis and plant-pathogen interactions.

**Conclusions:**

Our research provides comprehensive transcriptome information regarding SVBV infection in strawberry.

**Electronic supplementary material:**

The online version of this article (doi:10.1186/s12985-016-0584-5) contains supplementary material, which is available to authorized users.

## Background

*Strawberry vein banding virus* (SVBV) is one of the major viruses infecting strawberries. It has been reported worldwide, from North America to Australia to Belgium to Japan [1, 2]. In China, SVBV has been found in Henan, Hebei, Jilin and Zhejiang provinces causing serious loss of strawberry production [3]. Woodland strawberry infected with SVBV shows such characteristic symptoms as yellowing along the major leaf veins, shorter stolons, reduced plant growth and smaller fruit, as well as significant reduction in berry yield and quality. SVBV is transmitted either by grafting or by aphids in a semi-persistent manner [4]. SVBV is a member of the genus *Caulimovirus,* and has a circular dsDNA genome with one single stranded discontinuity on each DNA strand. The full-length of SVBV genome is ~8 kilobase pairs-long and encodes seven proteins [5].

Investigation of the pathogenic mechanism of SVBV in strawberry is important for better design of the disease control strategies. Woodland strawberry (family *Rosaceae*) is an important experimental plant for studying the mechanisms of virus-plant interactions and a facile model for investigating gene expression changes in response to pathogen infections [6, 7]. Because such response involves multiple physiological and metabolic processes, genome-wide expression profiling is a method of choice for studying these processes at the transcriptional level. Next generation sequencing techniques, such as RNA-Seq, have provided a powerful approach for studying the entire transcriptomes. RNA-Seq is used to measure gene expression, identify the mRNAs, non-coding RNAs and small RNAs, as well as to detect alternative splicing patterns [8].

Previous studies using RNA-Seq have greatly extended our understanding of the mechanisms underlying plant diseases caused by a wide range of pathogens. The transcriptome analysis of *Nicotiana tabacum* infected with *Cucumber mosaic virus* (CMV) suggested that, the changes in pigment metabolism pathway may be directly responsible for the disease development [9]. Comparative transcriptome analysis of two rice varieties in response to *Rice stripe virus* (RSV) infection showed that the lignification and cell wall strengthening play a critical role in resistance to RSV, providing a foundation for breeding for resistance to rice stripe disease [10]. Transcriptome gene expression analysis of *Chenopodium amaranticolor* in response to virus-induced hypersensitive response (HR) identified a number of candidate genes such as *RIN4*, *RPS2*, *PR1* and *COI1,* that are essential for a jasmonate-regulated defense. Each of these genes was specifically regulated during either TMV or CMV infection at both early and late stages of the HR [11].

In this study, we analyzed the response of strawberry plants to infection with SVBV using RNA-Seq and investigated the alterations in gene expression between the healthy and infected plants. We found that the genes involved in in many biological processes, such as plant pigment metabolism, photosynthesis and plant-pathogen interactions, were differentially expressed. These results will help to understand the regulatory mechanisms involved in strawberry response to SVBV infection.

## Results and discussion

### Illumina sequencing and reads assembly

**Fig 1:**
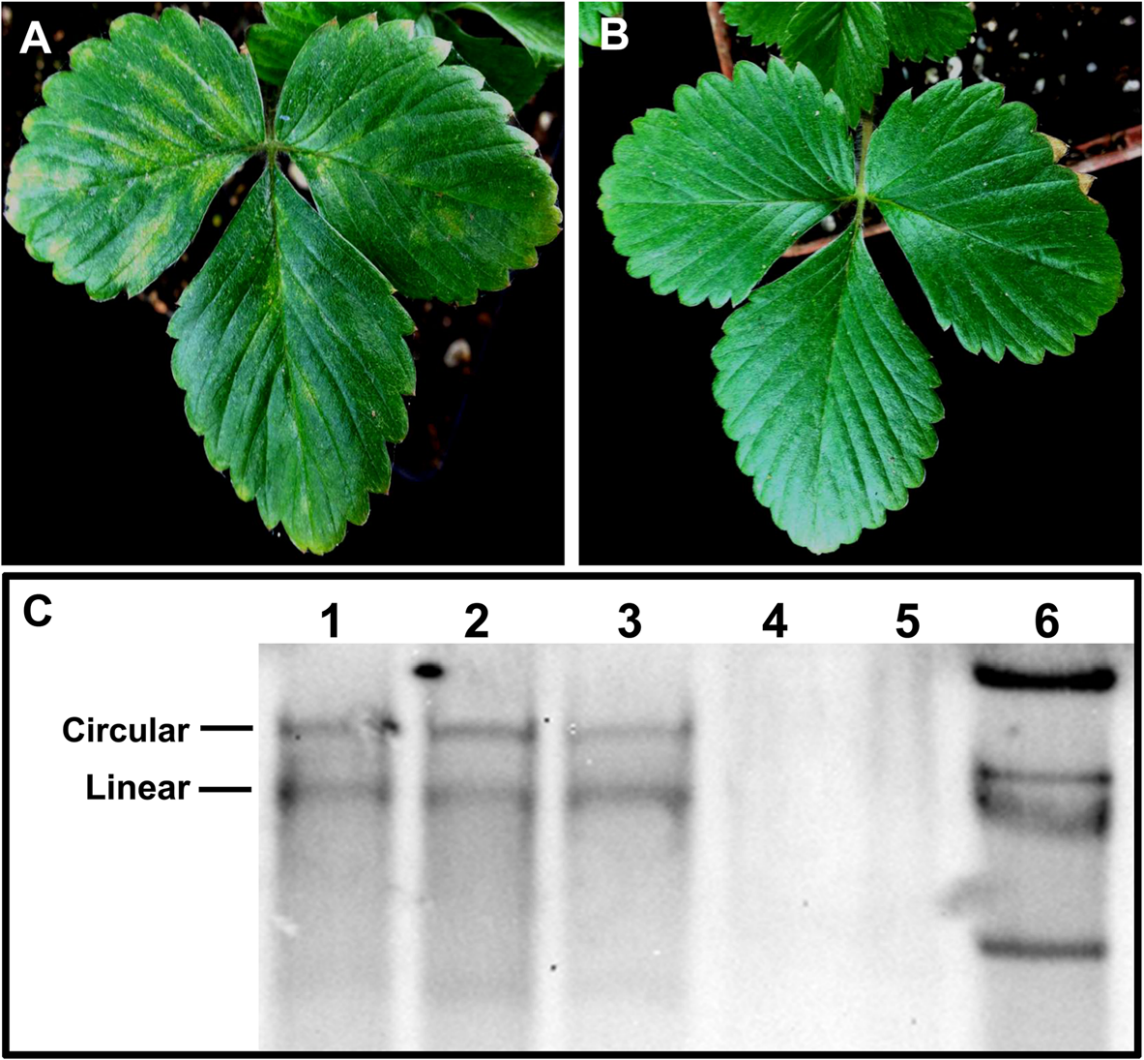
Symptoms of woodland strawberry. **a**: Vein banding symptom of woodland strawberry inoculated with SVBV infectious clone; (**b**): Symptomless woodland strawberry inoculated with *A. tumefaciens.* **c**: Genome DNA of SVBV detected by Southern blot. 1–3: Woodland strawberry inoculated with SVBV infectious clone; 4–5: Woodland strawberry inoculated with *A. tumefaciens*; 6: Plasmid pSVBV-E3

We chose strawberry plants infected with SVBV as a model to investigate host transcriptome responses. The inoculated strawberry leaves developed typical vein banding symptoms at 40 days post inoculation (Fig. 1a), while the mock-inoculated strawberry leaves did not show any symptoms (Fig. 1b). Southern blot analysis validated that symptomatic leaves were infected by SVBV (Fig. 1c). These leaves were collected for RNA extraction followed by Illumina sequencing. Two cDNA libraries, T1 (mock-inoculated control) and T2 (SVBV inoculated leaf material) were sequenced resulting in 76,157,614 and 72,701,140 sequence reads that encompassed 7.69 Gb and 7.34 Gb of sequence data, respectively. The reads’ length from both libraries ranged from 50 to 101 base pairs (bp) (Table 1). The tag density was sufficient for the quantitative analysis of gene expression. Sequence data from T1 and T2 libraries were combined to obtain 36,850 unigenes. The GC content of sequences from these was ~50 % with the phred quality score (Q30) above 90 %, indicating that the accuracy and quality of the sequencing data were sufficient for further analysis. The length distributions of total unigenes had similar patterns between the libraries, suggesting that there was no bias in the library construction (Additional file 2: Figure S1).

**Table 1:** Summary statistics for strawberry genes based on the RNA-Seq data

	T1	T2
Total reads	76157614	72701140
Sequence data	7.69 Gb	7.34 Gb
GC(%)	48.80	48.46
CycleQ30%	91.27	90.92
Mapped reads	63316297	60321762
UniqReads	62449329	59372555
MultiPosiReads	866968	949207
Total unigenes	18335	18515

### Global patterns of gene expression in response to SVBV infection

Further analysis revealed that 517 unigenes showed differential expression as compared to mock-inoculated control. Among these 517 differentially expressed genes (DEGs), 280 were up regulated, and 237 were down regulated. BLAST results were obtained for these DEGs against five protein databases, namely the non-redundant (nr, NCBI) protein database, Swiss-Prot database, Clusters of Orthologous Groups of proteins (COG, NCBI) database, the Gene Ontology (GO) database and Kyoto Encyclopedia of Genes and Genomes (KEGG) database. Among them, 498 DEGs had nr annotation, 388 DEGs had Swiss-Prot annotation, 195 DEGs had COG annotations, 425 DEGs had GO annotations and 79 DEGs had KEGG functional annotations.

**Fig 2:**
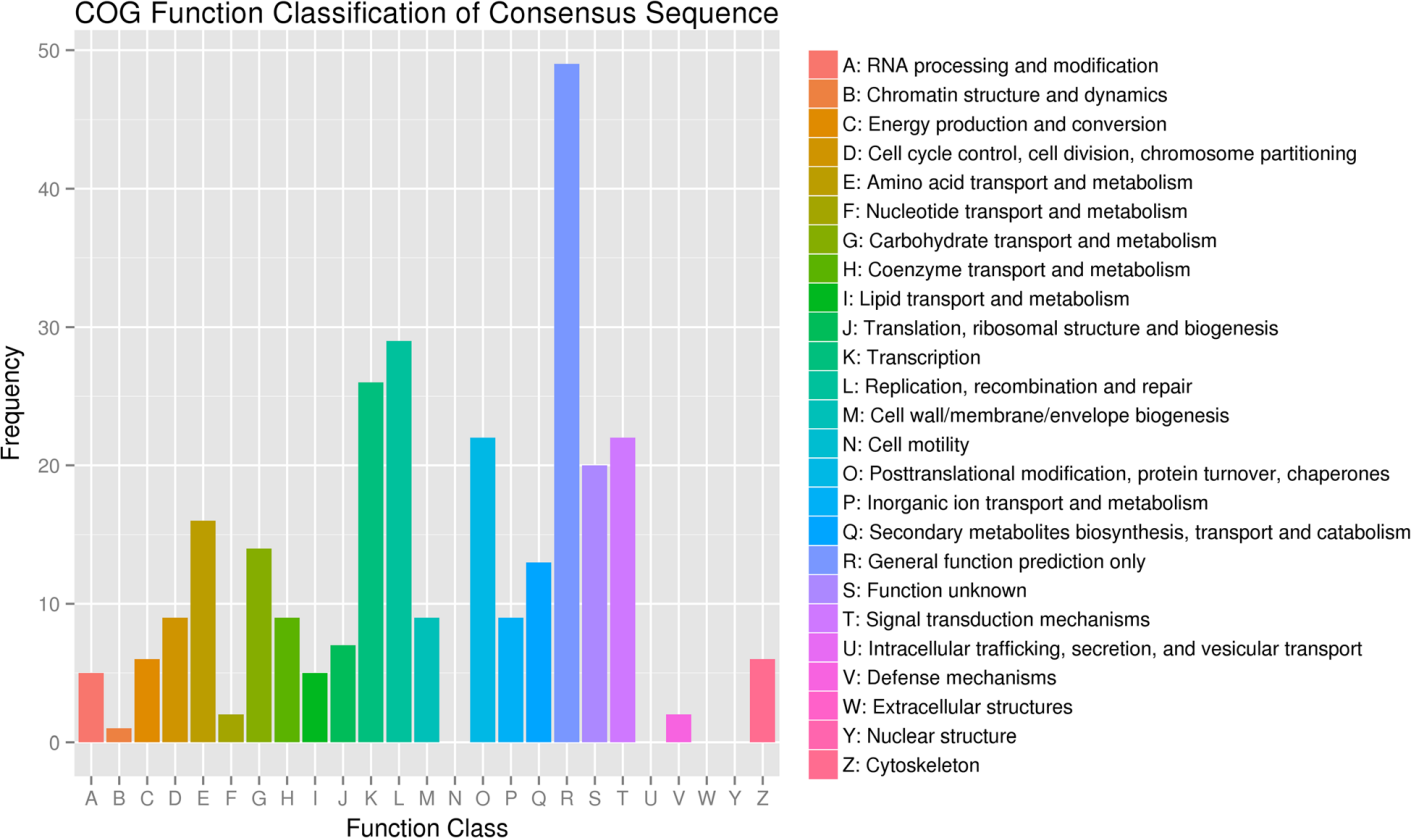
COG function Classification of DEGs. 195 DEGs were categorized into 21 functional groups. The x-axis shows the activity of DEGs and the y-axis shows the frequency of DEGs calculated in our library

**Fig 3:**
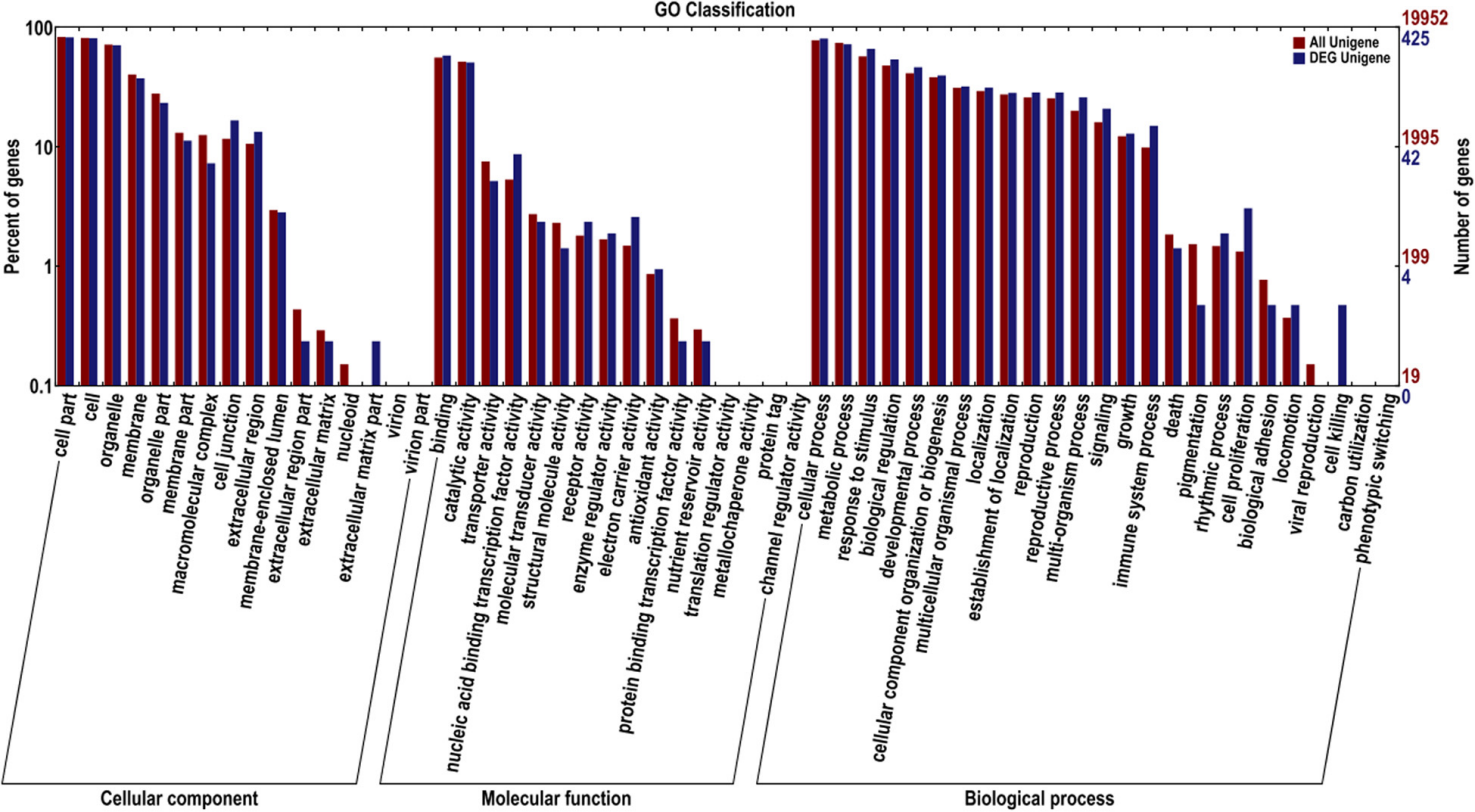
GO function Classification of DEGs. 425 DEGs were categorized into 47 functional groups. The x-axis shows the functional groups and the y-axis shows the percent of genes calculated in our library

Sequences with BLAST hits were further classified with COG, GO and KEGG pathway analysis. COG analysis indicated that 195 DEGs could be grouped into 21 clusters, and the DEGs mainly distributed in the cluster of ‘posttranslational modification, protein turnover and chaperones, replication, recombination, repair, transcription, and signal transduction mechanisms’. The cluster of ‘general function prediction’ accounted for 12 % of the total annotated DEGs, and their functions are still unknown (Fig. 2). The 425 DEGs annotated to the GO database were distributed in 47 functional types, including “growth and development”, “cell death”, “metabolism”, and “transcriptional regulation” (Fig. 3). Particularly, among these GO functional classes, a class of ‘cell killing’ has the largest differences between the SVBV-infected and virus-free samples, suggesting that the corresponding genes might be related to the pathogenic mechanism of SVBV. In conclusion, no matter which database we used, most of DEGs were putatively involved in cellular metabolism, signaling transduction, and plant-pathogen interactions. These genes appear to be relevant to the interactions between strawberry and SVBV.

### Analysis of important KEGG pathways

**Fig 4:**
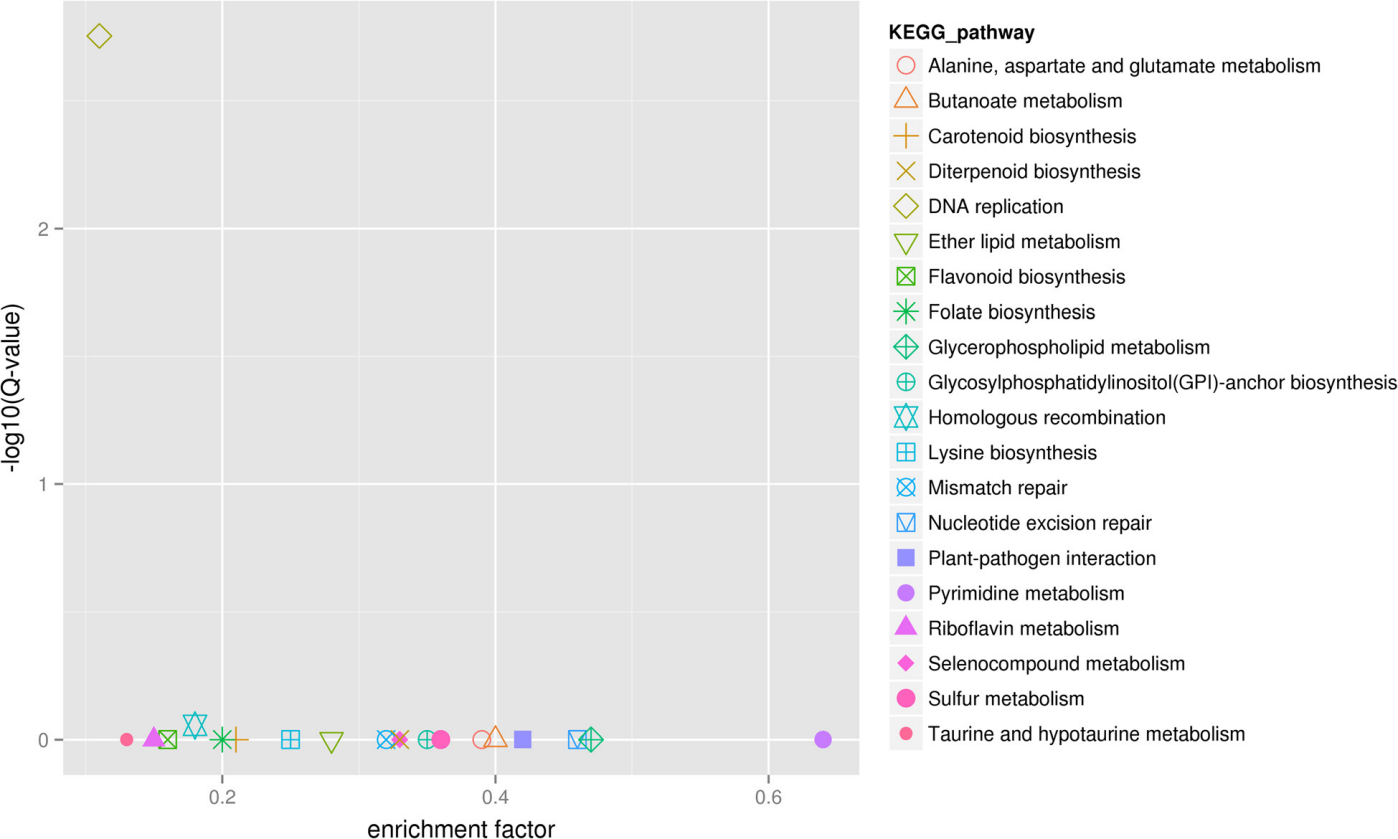
Important KEGG pathways influenced by SVBV infection. The x-axis shows the enrichment factors and y-axis shows the significance of enrichment of genes calculated in our library

**Fig 5:**
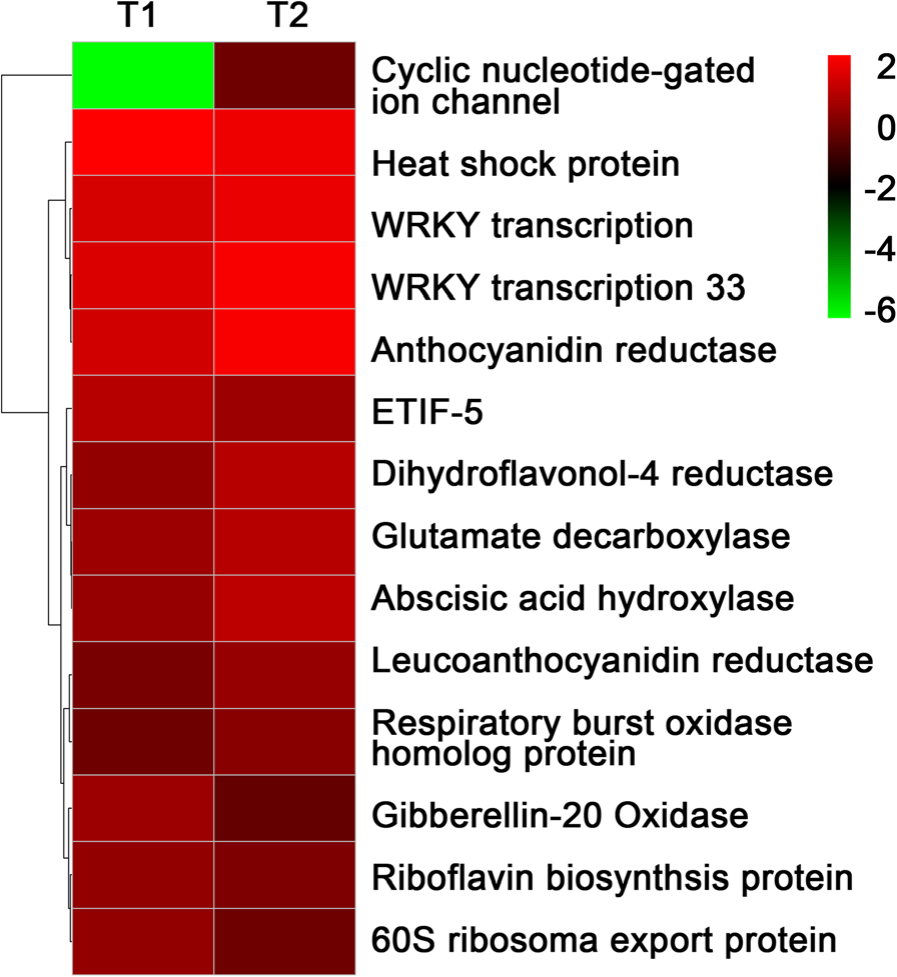
Expression patterns of 14 important genes related to SVBV pathogenicity. The two columns respectively show the expression of T1 and T2 from left to right, and every row represents a different gene. The color green, black and red indicate low, medium and high expression levels of genes, respectively

DEGs of strawberry infected with SVBV were annotated to 20 KEGG pathways (Fig. 4). The top three KEGG pathways containing the largest numbers of DEGs were DNA replication, plant-pathogen interactions and homologous recombination (Table 2). In addition, pigment metabolism and photosynthesis pathways also have a certain degree of enrichment. In these KEGG pathways, we found that the expressions of 14 genes were significantly altered (Fig. 5).

**Table 2:** Numbers of DEGs of top 18 KEGG pathways

Pathway	DEGs with pathway annotation (55)	Pathway ID	*P*-value
DNA replication	6 (10.9 %)	ko03030	3.26E-05
Plant-pathogen interaction	5 (9.09 %)	ko04626	0.016313
Homologous recombination	3 (5.45 %)	ko03440	0.038149
Pyrimidine metabolism	3 (5.45 %)	ko00240	0.054767
Glycerophospholipid metabolism	2 (3.63 %)	ko00564	0.062874
Cysteine and methionine metabolism	2 (3.63 %)	ko00270	0.124652
Carotenoid biosynthesis	2 (3.63 %)	ko00906	0.134477
Flavonoid biosynthesis	2 (3.63 %)	ko00941	0.139119
Mismatch repair	2 (3.63 %)	ko03430	0.180901
Starch and sucrose metabolism	2 (3.63 %)	ko00500	0.181124
Nucleotide excision repair	2 (3.63 %)	ko03420	0.221115
RNA transport	2 (3.63 %)	ko03013	0.235493
Alanine, aspartate and glutamate metabolism	2 (3.63 %)	ko00250	0.241640
Plant hormone signal transduction	2 (3.63 %)	ko04075	0.246704
Purine metabolism	2 (3.63 %)	ko00230	0.283547
Ubiquitin mediated proteolysis	2 (3.63 %)	ko04120	0.283547
Protein processing in endoplasmic reticulum	2 (3.63 %)	ko04141	0.295430
Glutathione metabolism	2 (3.63 %)	ko00480	0.303477

Glutamic acid decarboxylase (GAD) gene was significantly up-regulated by SVBV infection. GAD has been reported to be involved in “taurine and hypotaurine metabolism” of plant pigment metabolism and is the key enzyme in γ-aminobutyric acid (GABA) synthesis. In response to the biotic stress, plants rapidly increase GAD expression and accumulate quantity of GABA [12, 13], leading to reduce other free amino acid content and inhibiting the growth of stems [14]. The carotenoid biosynthesis pathway that mainly related to the photosynthesis also has a certain degree of enrichment, which might be highly associated with strawberry regulatory network in response to SVBV infection. The pathway might be highly associated with strawberry regulatory network in response to SVBV infection. Abscisic acid-8'-hydroxylase was related to “carotenoid biosynthesis” upon the pathogens and environmental stresses, the content of ABA caused by abscisic acid-8'-hydroxylase rise so rapidly, ABA depresses entire plant growth and vitro organs by functioning as a strong plant growth inhibitor [15].

Plant WRKY transcription factors and respiratory burst oxidase homolog protein were involved in plant-pathogen interaction, the up-regulation of factors is the embodiment of the plant immune responses [16]. Anthocyanin reductase and dihydroflavonol 4-reductase were both related to “flavonoid biosynthesis”. Anthocyanins, the plant secondary metabolites, play a key role in the process of fruit mature of strawberry [17].

### Confirmation of expression patterns by qRT-PCR

**Fig 6:**
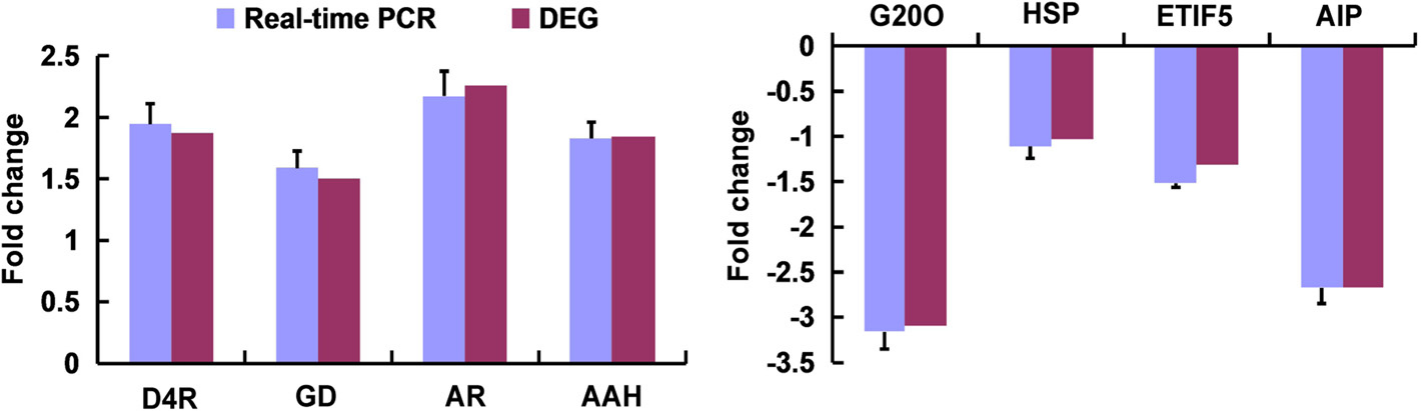
qRT-PCR validation of the relative expression levels of transcripts selected from the DGE analysis. Expression profiles of selected genes as determined by qRT-PCR (Red) and DGE (Blue). The signal intensity of each transcript was normalized using Actin. They axis shows the normalized expression level of the transcript, and the x axis shows the DEGs. Error bars represent the standard deviations of qRT-PCR (*n* = 3). D4H, Dihydroflavonol-4 reductase; GD, Glutamate decarboxylase; AR, anthocyanidin reductase; AAH, Abscisic acid hydroxylase; G20O, Gibberellin-20 Oxidase; HSP, heat shock protein; ETIF5, eukaryotic translation initiation factor-5; AIP, auxin-induced protein

In order to validate our DGE data, ten DEGs with annotations were selected for qRT-PCR analysis. Among them, eight out of ten DGEs exhibited a consistent expression pattern between RNA-Seq and qRT-PCR (Fig. 6). For example, both qRT-PCR and DGE analyses showed that genes encoding dihydroflavonol-4 reductase, glutamate decarboxylase, anthocyanidin reductase and abscisic acid hydroxylase were strongly up regulated in SVBV-infected strawberry compared to uninfected control. Analogously, the reduced expression of genes encoding the gibberellin 20-oxidase, heat shock protein 83 (Hsp83), eukaryotic translation initiation factor-5 (ETIF5) and auxin-induced protein 15 A revealed by RNA-Seq analysis were also validated by qRT-PCR. In addition, we analyzed expression of two genes encoding calmodulin and abscisic-acid receptor PYL4 by qRT-PCR. According to our data, neither of these two genes exhibited detectable differential expression during SVBV infection in the DEG dataset. Once again, qRT-PCR analysis validated these results. Thus, we have validated the conclusions of RNA-Seq analysis by independent qRT-PCR approach for all three major categories of genes: those up regulated, down regulated and unchanged in response to SVBV infection of strawberry.

## Materials and methods

### Sample preparation

Strawberry plants (*F. vesca*) were grown in a greenhouse on a cycle of 16 h light at 30 °C and 8 h dark at 25 °C. SVBV infectious clone, pBIN-1.5SVBV, constructed by insertion of 1.5-mer SVBV cDNA into the plant expression vector pBINPLUS were used for inoculation (unpublished data). *Agrobacterium tumefaciens* (EHA105) containing pBIN-1.5SVBV was inoculated onto the top two leaves of *Fragaria vesca* plants. For southern blot, total DNA was extracted from strawberry leaves, transferred onto a Hybond-N+ membrane (GE Healthcare), and immersed into the buffer containing radioactively labeled DNA. The radioactivity has been detected using X-ray film according to manufacturer’s instructions.

Mock-inoculated (T1) and SVBV-infected strawberry leaves (T2) were harvested at 40 dpi. In order to account for the variation between individual plants, three leaves from three different plants were used to prepare each RNA sample. Total RNAs were extracted from leaf tissues using TRIzol Reagent following the manufacturer’s instructions (Invitrogen). RNA concentration and integrity were analyzed on an Agilent 2100 Bioanalyzer (Agilent Technologies).

### Illumina sequencing and CDS analysis

cDNA library preparation and sequencing were conducted by the Biomarker Technology Company, Beijing, China. The cDNA library was sequenced on the Illumina Cluster Station and Illumina Genome Analyzer platform. The Trinity method was used for de novo assembly of reads. The transcripts were clustered by similarity of correct match length beyond the 80 % of longer transcripts or 90 % of shorter transcripts using the multiple sequence alignment tool BLAST [18]. The raw sequence data of two samples were uploaded to NCBI (http://trace.ncbi.nlm.nih.gov/Traces/sra_sub/sub.cgi), and the accession numbers are SRR1930099 for SVBV-infected sample and SRR1930097 for mock-inoculated control, respectively.

The coding sequences (CDS) of the unigenes were predicted by ‘getorf’ model of EMBOSS (http://emboss.sourceforge.net/apps/cvs/emboss/apps/getorf.html/). The complete CDS sequences were compared with the CDS sequences of Nipponbare (Os-Nipponbare-Reference-IRGSP-1.0) (http://rapdb.dna.affrc.go.jp/download/irgsp1.html).

### Functional annotation and digital gene expression analysis

We annotated unigenes based on a set of sequential BLAST searches designed to find the most descriptive annotation for each sequence [19]. The assembled unigenes were searched against sequences stored in the online databases NR, NT, Swiss-Prot, KEGG, COG and GO. The Blast2GO program was used to obtain GO annotations for the unigenes, and WEGO software was then used for GO functional classification [20].

Gene expression levels were measured in RNA-Seq (Invitrogen) analyses as numbers of reads and were normalized with RPKM [21]. IDEG6 software was used to identify differentially expressed genes in pair-wise comparison [22], and the results of all statistical tests were corrected for multiple testing with the Benjamini–Hochberg false discovery rate (FDR < 0.01).

### Quantitative RT-PCR (qRT-PCR) analysis

To validate the results of pyrosequencing analysis, we determined the expression levels of 10 DEGs by qRT-PCR. Total RNAs from each sample were extracted using TRIzol reagent (Invitrogen) and qRT-PCR was performed using SYBR® *Premix Ex Tap*™ II (TaKaRa) according to the manufacturer’s instructions. qRT-PCR cycles were carried out on a Step One Real-Time PCR system (ABI) as follows: 30 s at 95 °C for denaturation, followed by 40 cycles of 5 s at 95 °C for denaturation, 30 s at 60 °C for annealing. Fluorescence data was collected at 60 °C. A melting curve was performed from 60 °C to 95 °C (held for 1 s per 0.1 °C increase) to examine the specificity of the amplified product. Primers used in qRT-PCR for validation of differentially expressed genes are shown in Additional file 1: Table S1, an *actin* gene from woodland strawberry was selected as the reference gene. Expression quantification and data analysis were performed in accordance with Bustin et al. [23].

## Additional files

Additional file 1: Table S1. Primers used for qRT-PCR validation of DGEs. (DOC 30 kb) Additional file 2: Figure S1. Length distribution of unigenes in the assembled transcriptomes. The x-axis shows the lengths of unigenes and the y-axis shows the number of unigenes calculated in our library. (TIF 462 kb)
