# Supplementary material for: Transcriptome analysis of woodland strawberry (Fragaria vesca) response to the infection by Strawberry vein banding virus (SVBV)
Source: Virol J. 2016 Jul 13;13:128. doi: 10.1186/s12985-016-0584-5 (PMC4942977; doi:10.1186/s12985-016-0584-5)
Supplement: Additional file 1: Table S1. — Primers used for qRT-PCR validation of DGEs. (DOC 30 kb) [file 12985_2016_584_MOESM1_ESM.doc]

**Table 1** Primers used for qRT-PCR validation of DGEs.

| Gene ID | Foreword primer | Reverse primer |
| --- | --- | --- |
| Dihydroflavonol-4 reductase | ACAAGGAATGAGCTACACAG | TATGAGTTCGGTCCCTGCAG |
| Glutamate decarboxylase | CTGAGAAGCTAGTGAAGGAG | GATGGTGCTCTAGCATGTG |
| Anthocyanidin reductase | CCTACCAAGGCAAGATAGC | ACGTCGACCCATCACCGAC |
| Abscisic acid hydroxylase | ACATTGAATGCAAATTACAAG | CATCCAGCAGTCGCCTATC |
| Gibberellin-20 oxidase | GCTGCCGACTCCAACTCCGAC | TCAAGTCTTCGTGGCTGAGC |
| Heat shock protein | GGAGATCTGGATCTTCAG | GGACCTCGACGCCAACAG |
| ETIF5 | CCTAACAACAGTTGTGGCAG | AACAAATCCCAGGACTCTG |
| Auxin-induced protein | GCAAACGAGAAGGTTCTAG | CCGATGTTTCTCCCTCTG |
| Actin | GTGGAAACATCACTGACTG | ATTCAAATTCAACTCACCAG |
