# Supplementary figures and images for: Transcriptome analysis of woodland strawberry (Fragaria vesca) response to the infection by Strawberry vein banding virus (SVBV)
Source: Virol J. 2016 Jul 13;13:128. doi: 10.1186/s12985-016-0584-5 (PMC4942977; doi:10.1186/s12985-016-0584-5)

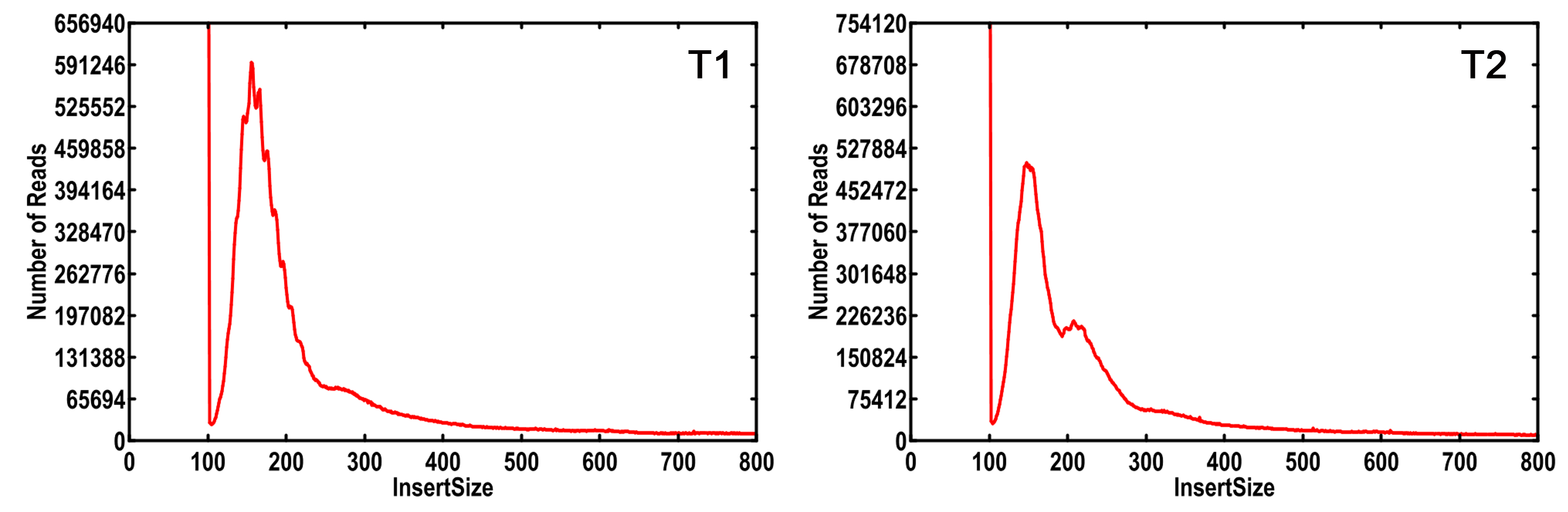

Supplement: Additional file 2: Figure S1. — Length distribution of unigenes in the assembled transcriptomes. The x-axis shows the lengths of unigenes and the y-axis shows the number of unigenes calculated in our library. (TIF 462 kb) [file 12985_2016_584_MOESM2_ESM.tif]
